# Effect of food hardness on chewing behavior in children

**DOI:** 10.1007/s00784-020-03425-y

**Published:** 2020-06-29

**Authors:** Nabeel Almotairy, Abhishek Kumar, Anastasios Grigoriadis

**Affiliations:** 1 Unit of Oral Rehabilitation, Division of Oral Diagnostics and Rehabilitation, Department of Dental Medicine, Karolinska Institutet, Box 4064, Alfred Nobels Allé 8, 141 04 Huddinge, Sweden; 2 Department of Orthodontics and Pediatric Dentistry, College of Dentistry, Qassim University, Buraidah, Saudi Arabia

**Keywords:** Jaw kinematics, Jaw muscle activity, Mastication, Viscoelastic food, Child, Development

## Abstract

**Objective:**

To investigate the effects of food hardness on chewing behavior in children compared with adults.

**Materials and methods:**

Healthy children (3–17 years) were equally divided into five groups based on their dental eruption stages. Each participant ate soft and hard viscoelastic test food models (3 each), while the three-dimensional jaw movements and electromyographic (EMG) activity of the bilateral masseter muscles were recorded. The data from the children were compared with a control group of healthy adults (18–35 years). The data were analyzed with nonparametric tests.

**Results:**

There was no significant difference in the number of chewing cycles and the duration of the chewing sequence between children groups and adults. Children with primary dentition (3–5 years) showed shorter lateral jaw movement and higher muscle activity at the end of the chewing sequence, compared with adults. Further, children’s age-groups (3–14 years) failed to adapt their jaw muscle activity to food hardness. However, at the late-permanent dentition stage (15–17 years), children were capable of performing adult-like chewing behavior.

**Conclusions:**

Overall, it seems that children as young as 3-year-old are quite competent in performing basic chewing function similar to adults. Yet, there are differences in the anticipation or adaption of jaw muscle activity and jaw kinematics to food hardness.

**Clinical relevance:**

The study may have clinical implication in the diagnosis and management of children with chewing impairment associated with dental malocclusions and other orofacial dysfunctions.

## Introduction

Chewing behavior can be stated as the complex and dynamic action of cutting the food and preparing it for swallowing. Previous studies have indicated that chewing behavior is closely related to the functional state of the mouth and teeth (for review, see [1]). Normal chewing behavior is integral in enhancing the taste of the food, initiating the process of digestion, stimulation of saliva secretion, and facilitating the safe-swallow process [2]. Studies have shown that compromised oral health, either due to infection or impaired chewing function, is an essential determinant of nutrition [3]. Impaired chewing function substantially influences the food eating habits [4] and the supply of key ingredients required for maintaining bodily functions and oral health-related quality of life [5].

Chewing movements are initiated by specialized neural circuits in the brain stem called the central pattern generator [6]. The chewing movements can also be initiated by the primary motor cortex and the primary somatosensory cortex [7]. The regulation and fine-tuning of the chewing movements, however, require sensory feedback from several mechanoreceptors impeded in different orofacial structures such as the periodontium, masticatory muscle, and temporomandibular joint [8]. The central nervous system, thus, assimilates and integrates the sensory inputs obtained from these receptors, which are used to regulate the chewing movements. The sensorimotor regulation of the chewing movements has been described in detail in adults [9–15]. It has been shown in a series of well-controlled studies that the jaw muscle activity and jaw kinematics during chewing adapt to the changes in food hardness [9–15] and to the changing mechanical properties of the bolus [10]. Aging was associated with a decline in masticatory muscle mass, reduced bite forces, and reduced salivary flow rate and the number of oral sensory receptors (for review [14, 16]). However, it has little effect on the ability to pulverize the food bolus into smaller pieces in healthy old adults with good oral state [14, 15]. It was suggested that these people maintained their chewing ability by increasing the number of chewing cycles and adapted their jaw kinematics and muscle activity to the increased food hardness [14, 15].

Growing children exhibit substantial morphological changes in the orofacial structures responsible for the act of chewing. The changes in the orofacial structures may exert a substantial challenge in the sensorimotor regulation of the chewing behavior. Thus, it may indicate that growing children should learn and adapt their chewing behavior in response to the changes in the orofacial structures. In our previous work, we comprehensively reviewed the age-related changes in jaw sensorimotor regulation and objective parameters of chewing in healthy children [17]. Consequently, it was shown that chewing parameters such as maximum bite force, jaw muscle activity, and jaw kinematics gradually change with the development of the orofacial structures and were mainly influenced by the dentition status [5]. Notably, studies on jaw kinematics during chewing showed that children with primary dentition exhibit shorter and broader jaw trajectories in comparison with adults [18]. A simple “meta-analysis” suggested a transition to an “adult-like” regulation of bite forces, jaw kinematics, and jaw muscle activity during the late-mixed to early-permanent dentition stages. We, therefore, hypothesize that the chewing behavior in children will show age-related changes, where a shift to an adult-like chewing behavior will occur during the late-mixed to early-permanent dentition stages. We also hypothesize that young, healthy children will show signs of difficulty in regulating their jaw muscle activity in relation to the food hardness as compared with adults. This study aims to investigate the effects of food hardness on the jaw kinematics and jaw muscle activity during the chewing sequence in children compared with adults.

## Material and methods

### Participants

The present study involved human participants and was conducted in accordance with the ethical standards of the institutional and/or national research committee and with the 1964 Helsinki declaration and its later amendments or comparable ethical standards. This study was approved by the Swedish Ethical Review Authority**,** Stockholm, Sweden (Dnr: 2018/726–31/2). The experiment was introduced to children with their parents/legal guardians who came to regular dental check-ups in the Pedodontics Specialist Clinics at Karolinska Institutet, Sweden. The sample size was calculated prior to the study based on previous studies of similar behavioral tasks [9, 11–13] using G*Power software (version 3.1; Heinrich Heine University Düsseldorf, Düsseldorf, Germany). For 95% confidence level (*α* = 0.05; *β* = 80%) and effect size of 0.45, a sample of 13 participants is needed per group. However, among the seventy-two healthy children (3–17 years) that were recruited for this study, fifty children performed the task (see limitations). The participating children were categorized into five age-groups based on their dental eruption stages (primary dentition; early-mixed dentition; late-mixed-dentition; early-permanent; late-permanent). The five children age-groups were compared with a control group of 10 healthy adults (Table 1). All the participants had average general health and body mass index with no known systematic health conditions or painful disorders. Further, the participants were free from active dental caries, history of dental trauma, restorations or crowns, moderate to severe malocclusion, active orthodontic treatment, or fixed retainers. Written informed consent was obtained before the start of the experiment from all the participants or, if the participants are under 18 years, from a parent and/or legal guardian.

**Table 1:** Shows the number and age of the participating children and adults (boys/men *♂;* girls/women *♀*)

#	Group*	Number of participants	Age range (years)	Mean age (years)	SD
1	Primary dentition	10 (4 ♂; 6 ♀)	3.0–5.9	5.3	0.3
2	Early-mixed dentition	10 (5 ♂; 5 ♀)	6.0–8.9	8.0	0.7
3	Late-mixed dentition	10 (8 ♂; 2 ♀)	9.0–11.9	11.0	0.8
4	Early-permanent dentition	10 (4 ♂; 6 ♀)	12.0–14.9	13.2	0.9
5	Late-permanent dentition	10 (8 ♂; 2 ♀)	15.0–17.9	16.3	0.7
6	Adult control	10 (6 ♂; 4 ♀)	18.0–35.0	25.9	4.1

*Group classification criteria:

Group 1: defined as when the primary dentition is completed

Group 2: defined as when the permanent first molars and permanent incisors erupted in the mouth

Group 3: defined as when either the permanent first and second premolars and canines erupted in the mouth

Group 4: defined as when the eruption of all the permanent teeth is completed except the permanent second and third molars

Group 5: defined as when the all the permanent teeth except third molars are present in the mouth

Group 6: defined as when the eruption of all the permanent teeth is completed

### Recording jaw movements and muscle activity

**Fig. 1:**
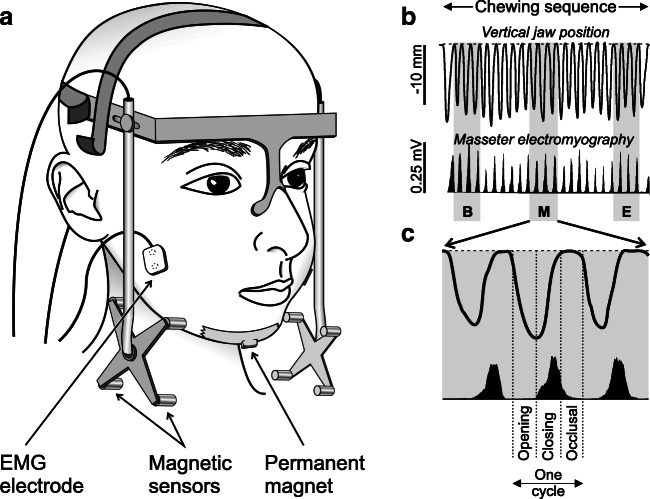
A Graphic depiction of the apparatus setup (**a**) which consists of customized jaw tracker equipped with bilateral four magnetic sensors. A permanent magnet was secured below the chin of each participant using a tissue-friendly adhesive tape. In addition, the masseter EMG signals were recorded bilaterally using custom-made bipolar electrodes. (**b**) Shows an example of a chewing sequence of vertical jaw movement and masseter muscle EMG activity. The whole chewing sequence was divided into three segments (beginning, middle, and end (gray areas)). (**c**) Shows a close-up view of the middle chewing segment where individual chewing cycles were divided into three chewing phases: an opening phase where the jaw opens 1 mm or more from the occlusal state and ends at the peak jaw opening; a closing phase when the jaw returned to the same vertical position where the jaw opening phase began, and an occlusal phase which starts at the end of the closing phase and ends when the opening phase of the next cycle begins

The detailed specifications of the equipment used in the current study were reported in our previous publications [9, 11–13]. Briefly, the three-dimensional movements of the lower jaw in reference to the upper jaw were measured using a custom-built apparatus (Umeå University, Physiology Section, IMB, Umeå, Sweden). A small magnet (10 × 5 × 5 mm) was secured below the chin by a tissue-friendly tape (Leukoplast® adhesive tape). A lightweight frame equipped with an array of multiple magnetic sensors (accuracy: 0.1 mm; bandwidth: 0–100 Hz) was attached to the participant’s head in a spectacle-like manner to track in three dimensions the position of the magnet. The frame was strapped to the head (Fig. 1a). Bipolar surface electromyography (EMG) was used to record the bilateral masseter muscle activity (diameter: 2 mm; 12 mm apart; bandwidth: 6 Hz–2.5 kHz). Each participant was asked to clench 2–3 times to palpate and locate the prominent (central) part of the masseter muscle. The electrodes were then placed over the skin in parallel to the direction of the muscle fibers [19, 20]. Before the placement of the electrodes, an electrode gel was applied, and the skin over the masseter muscle was gently cleansed with alcohol. The data was acquired and stored offline and analyzed with customized software (SC/Zoom, Umeå University, Physiology Section, IMB, Umeå, Sweden).

### Experimental protocol and procedure

The edible elastic food models were produced in our laboratory following the recipe described in previous studies [9, 10] in order to optimize the control of the rheological properties of the food, and thus avoiding variations in the measured parameters caused by food inconsistencies. Accordingly, two grades of gelatin were used: one with 25 g of 150 bloom (soft) and one with 41.5 g of 250 bloom (hard). The two grades were mixed with 132 g glucose, 111 g sugar, 84 g water, and citric acid. To distinguish the two types of food, the food was colored yellow for soft food and green for hard food. The food was prepared under a water path at 80 °C for 2 and 4 h for the soft and hard food, respectively. The mixtures were then poured into cylindrical Plexiglas models for 24 h and in an airtight box for 72 h. Each food model was 10 mm high and 20 mm in diameter.

The volunteers participated in a single experimental session. Before the start of the experiment, the participating children were shown a video clip of a child demonstrating the chewing task. During the experiment, each participant was asked to chew and swallow the soft and hard viscoelastic test food models (3 trials each). The participants were asked to choose their preferred chewing side and were instructed to chew only on that side throughout the experiment (i.e., the chewing side). The test food was presented to participants in random order. Before each trial, the test food was visually concealed from the participants’ view and placed on the extended tongue of the participants. Then, the participants were instructed to close their teeth into intercuspation and keep the test food between the tongue and the palate. After 2–4 s, the participants were signaled to chew the test food and were instructed to close back their teeth into intercuspation once they have finished chewing and swallowing the food. Between the trials, the participants were free to rest, speak, drink water, and rinse their mouth.

### Data analysis

The entire chewing sequence for each food model was divided into three segments: beginning, middle, and end (Fig. 1b). Each of these segments was the average of three consecutive chewing cycles. Each chewing cycle was divided into a jaw opening phase, jaw-closing phase, and occlusal phase (Fig. 1c). The jaw opening phase starts when the jaw opens 1 mm or more from the occlusal state and ends at the peak of the jaw opening. The jaw-closing phase starts when the jaw returns to the same vertical position where the jaw opening phase began, while the jaw occlusal phase starts at the end of the closing phase and ends when the opening phase of the next cycle begins.

The EMG signals were sampled at 3.2 kHz and processed by a root mean square (± 31 ms moving window). The EMG signals were time-varying between the participants. Therefore, the time-variable EMG signals were normalized while preserving the temporal information for each phase by dividing the EMG signals in each phase to the average activity during all the chewing cycles for each participant (see [9]). The EMG activity of the masseter muscle between the chewing side versus the non-chewing side showed no statistical differences for each age-group (*p* > 0.05). Therefore, the EMG activity obtained from the chewing and non-chewing sides was integrated and used for analysis in the current study.

### Statistical analysis

The outcome parameters of the current study are described as chewing sequence, jaw kinematics, and EMG activity of the masseter muscle. The chewing sequence is described in terms of the number of chewing cycles, chewing sequence duration, and chewing rate. While the jaw kinematics are described in terms of the jaw opening and closing velocity, vertical and lateral amplitude of the jaw movement, and the duration of the jaw opening, closing, and occlusal phases. Similarly, the EMG activity of the masseter muscle was described in terms of root mean square values during the jaw-closing and occlusal phases. The data from the abovementioned parameters were assessed with the Shapiro-Wilks test and histogram plots. The data appeared to violate the assumption of data normal distribution. Hence, nonparametric tests were used to analyze between-group (age-group) differences and within-group (i.e., food: hard/soft and chewing sequence: beginning/middle/end) differences.

Accordingly, all the abovementioned parameters were subjected to the Kruskal–Wallis H test to analyze between-group differences with pairwise post hoc comparison using Dunn-Bonferroni correction. Further, the Wilcoxon Matched Pairs Test was used to analyze the differences between the soft and hard food at the beginning of the chewing sequence for each of the outcome parameters within each group. Friedman ANOVA and Kendall Coefficient of Concordance test were used to analyzing the differences between the three segments of the chewing sequence (beginning, middle, and end) of each of the outcome parameters within each age-group. The tests’ significance level was marked at a *p* value of less than 0.05.

## Results

Sixty participants (50 children and 10 adults) were recruited and were equally divided into six age-groups (Table 1). The median (interquartile range) of the outcome parameters of the chewing behavior obtained from the children and adults are presented in Table 2. The major findings of each outcome variable were presented below (please refer to Table 3 for detailed statistical results). For each of the outcome parameters, we have first presented the results of between-group differences, followed by food differences (soft and hard food) within each group. Then, the results are presented for the progression of chewing sequence (beginning, middle, and end) while eating the soft or the hard food within each group.

**Table 2:** Median (interquartile range) of all the outcome parameters obtained during the chewing of soft and hard viscoelastic test food models performed by the participating children and adults

Variable	Food	Segment	Primary	Early-mixed	Late-mixed	Early-permanent	Late-permanent	Adults
Chewing cycle (n)	Soft	ــ	22.67 (17.75–26)	20 (17.75–24)	20.67 (19.67–28.92)	17.33 (12.67–19.58)	19.33 (17.5–26.67)	19.83 (16.67–24.75)
	Hard	ــ	26.5 (20.42–36.33)	18.33 (15.25–22.08)	22 (18.25–34.42)	20.5 (11.17–25)	23.17 (17.5–24.83)	24.83 (22.42–31.75)
Chewing duration (s)	Soft	ــ	15.63 (12.37–18.40)	13.22 (11.49–15.65)	13.77 (10.56–17.38)	10.58 (8.93–13.02)	15.74 (14.61–17.83)	13.32 (11.32–19.86)
	Hard	ــ	17.24 (11.20–23.59)	12.24 (9.89–15.83)	11.70 (10.97–17.28)	11.60 (7.30–17.59)	14.55 (12.45–18.85)	18.18 (13.51–23.61)
Chewing rate (Hz)	Soft	ــ	1.43 (1.24–1.58)	1.44 (1.31–1.68)	1.67 (1.56–1.88)	1.55 (1.35–1.72)	1.31 (1.21–1.55)	1.51 (1.28–1.59)
	Hard	ــ	1.60 (1.57–1.64)	1.52 (1.34–1.61)	1.74 (1.69–1.87)	1.62 (1.48–1.73)	1.40 (1.26–1.67)	1.51 (1.28–1.63)
Vertical amplitude (mm)	Soft	Begin	11.93 (9.38–12.87)	12.35 (11.38–16.98)	11.24 (10.69–12.53)	13.37 (10.95–15.12)	11.47 (9.32–13.01)	13.48 (10.39–17.85)
		Middle	10.84 (8.99–11.95)	11.30 (9.83–15.75)	10.52 (8.87–12.62)	11.96 (11.19–12.93)	11.19 (9.69–13.19)	11.91 (9.36–15.94)
		End	9.22 (7.07–11.67)	10.25 (9.17–14.79)	9.32 (8.46–10.16)	10.78 (9.59–11.96)	10.88 (8.138–11.60)	11.73 (8.31–13.59)
	Hard	Begin	12.08 (10.64–14.52)	12.77 (11.97–16.75)	11.31 (10.50–13.07)	12.83 (11.09–15.78)	12.37 (11.21–14.56)	14.11 (10.92–18.29)
		Middle	10.47 (7.89–12.97)	11.46 (10.41–14.87)	10.95 (9.10–12.53)	12.747 (10.09–14.40)	11.52 (10.15–12.54)	13.63 (9.67–16.49)
		End	9.97 (7.29–10.78)	11.29 (9.98–13.89)	9.66 (7.87–11.32)	10.77 (9.86–12.69)	10.07 (7.99–12.03)	11.53 (8.16–14.08)
Lateral amplitude (mm)	Soft	Begin	4.89 (4.01–5.59)	7.19 (5.58–7.92)	6.47 (4.98–8.39)	6.64 (6.11–7.96)	6.60 (6.25–9.25)	8.45 (7.15–9.04)
		Middle	4.98 (4.72–5.12)	7.17 (6.31–7.44)	6.76 (5.37–7.02)	6.70 (5.76–7.04)	7.04 (5.60–9.31)	8.45 (6.99–9.23)
		End	4.56 (3.61–5.12)	6.63 (5.73–7.10)	5.29 (4.93–7.07)	6.08 (5.39–6.75)	5.35 (4.65–7.78)	7.49 (6.27–8.18)
	Hard	Begin	4.47 (3.73–5.58)	7.53 (5.88–7.94)	6.31 (5.36–7.22)	7.26 (6.89–8.79)	7.36 (6.27–9.89)	8.36 (7.37–10.35)
		Middle	4.82 (3.76–5.47)	7.51 (6–7.83)	6.21 (5.76–7.37)	7.23 (6.39–8.16)	6.88 (5.76–9.49)	8.80 (6.70–9.71)
		End	3.77 (3.25–4.72)	7.19 (6.64–7.63)	4.98 (4.65–6.69)	6.67 (5.20–7.22)	5.64 (4.94–7.06)	7.18 (5.51–8.76)
Jaw opening velocity (mm/s)	Soft	Begin	65.40 (49.31–83.91)	68.30 (65.53–70.58)	56.83 (54.60–75.60)	70.36 (61.72–77.48)	60.19 (56.93–70.23)	62.71 (48.66–110.47)
		Middle	64.30 (48.62–79.84)	61.8 (59.91–72.26)	58.42 (47.90–73.61)	65.22 (51.41–72.51)	57.76 (45.19–75.37)	54.52 (43.01–94.20)
		End	52.63 (33.28–60.37)	57.79 (49.78–73.15)	47.96 (43.56–58.65)	53.01 (46.04–63.38)	49.90 (34.38–54.48)	51.90 (42.04–62.23)
	Hard	Begin	84.38 (59.42–100.84)	69.14 (64.53–76.56)	64.75 (58.87–82.98)	69.69 (59.40–71.56)	71.42 (64.13–85.26)	72.53 (50.23–114.79)
		Middle	68.93 (51.89–74.43)	66.51 (60.37–73.19)	62.29 (52.05–75.45)	64.19 (59.63–72.67)	62.95 (55.43–74.85)	66.35 (51.57–111.62)
		End	66.28 (40.55–71.5)	61.95 (53.08–70.27)	55.22 (43.45–65.06)	60.05 (52.92–65.69)	45.30 (35.05–65.57)	50.89 (46.25–62.79)
Jaw-closing velocity (mm/s)	Soft	Begin	53.05 (41.73–68.86)	69.30 (56.28–88.67)	62.74 (51.56–71.39)	77.67 (48.51–81.18)	56.97 (51.14–61.78)	69.84 (44.55–110.33)
		Middle	52.54 (44.34–75.39)	58.36 (52.08–73.36)	61.58 (47.60–67.35)	58.53 (52.18–71.72)	59.64 (55.90–63.03)	64.69 (46.50–98.87)
		End	46.66 (28.31–65.70)	48.27 (44.66–69.36)	48.13 (40.42–54.12)	47.66 (37.42–60.39)	49.57 (42.89–52.11)	63.93 (40.14–79.19)
	Hard	Begin	60.72 (53.18–83.96)	74.97 (58.86–93.97)	66.60 (57.64–76.84)	65.41 (54.52–79.85)	72.98 (66.14–79.50)	73.97 (47.26–112.68)
		Middle	62.53 (47.36–68.21)	64.70 (53.90–77.08)	62.61 (54.602–78.22)	62.35 (53.24–72.56)	64.29 (55.51–70.95)	77.69 (51.25–111.83)
		End	49.42 (33.97–56.26)	55.83 (49.11–69.76)	52.07 (41.29–57.50)	54.17 (47.27–58.01)	46.52 (41.75–51.50)	60.65 (37.46–72.37)
Occlusal duration (s)	Soft	Begin	0.25 (0.21–0.31)	0.26 (0.24–0.29)	0.22 (0.20–0.27)	0.23 (0.20–0.27)	0.32 (0.26–0.37)	0.26 (0.23–0.28)
		Middle	0.28 (0.22–0.34)	0.27 (0.22–0.30)	0.22 (0.20–0.28)	0.26 (0.20–0.32)	0.30 (0.27–0.34)	0.29 (0.23–0.32)
		End	0.33 (0.26–0.44)	0.26 (0.23–0.30)	0.23 (0.23–0.28)	0.23 (0.21–0.30)	0.30 (0.25–0.34)	0.30 (0.25–0.33)
	Hard	Begin	0.23 (0.20–0.25)	0.24 (0.22–0.27)	0.22 (0.20–0.25)	0.24 (0.18–0.28)	0.28 (0.27–0.34)	0.28 (0.21–0.31)
		Middle	0.27 (0.229–0.30)	0.26 (0.24–0.30)	0.23 (0.20–0.25)	0.24 (0.22–0.31)	0.28 (0.26–0.30)	0.28 (0.24–0.34)
		End	0.29 (0.28–0.53)	0.28 (0.27–0.31)	0.24 (0.24–0.27)	0.26 (0.21–0.30)	0.29 (0.26–0.35)	0.27 (0.23–0.36)
Opening duration (s)	Soft	Begin	0.20 (0.16–0.26)	0.23 (0.18–0.27)	0.21 (0.17–0.22)	0.23 (0.20–0.29)	0.21 (0.19–0.27)	0.26 (0.21–0.28)
		Middle	0.24 (0.19–0.29)	0.24 (0.22–0.26)	0.19 (0.16–0.24)	0.19 (0.18–0.25)	0.22 (0.20–0.28)	0.24 (0.20–0.27)
		End	0.21 (0.17–0.27)	0.22 (0.18–0.25)	0.22 (0.18–0.27)	0.23 (0.20–0.28)	0.24 (0.20–0.27)	0.28 (0.24–0.34)
	Hard	Begin	0.16 (0.14–0.17)	0.22 (0.18–0.27)	0.18 (0.16–0.22)	0.22 (0.20–0.29)	0.20 (0.17–0.26)	0.23 (0.20–0.27)
		Middle	0.17 (0.15–0.21)	0.21 (0.19–0.25)	0.17 (0.17–0.19)	0.18 (0.17–0.25)	0.19 (0.16–0.22)	0.22 (0.18–0.25)
		End	0.16 (0.15–0.19)	0.21 (0.21–0.23)	0.18 (0.16–0.26)	0.21 (0.20–0.22)	0.23 (0.22–0.26)	0.26 (0.21–0.31)
Closing duration (s)	Soft	Begin	0.24 (0.20–0.28)	0.25 (0.22–0.27)	0.21 (0.17–0.23)	0.21 (0.19–0.25)	0.24 (0.22–0.26)	0.25 (0.20–0.27)
		Middle	0.21 (0.17–0.24)	0.22 (0.21–0.25)	0.17 (0.15–0.20)	0.18 (0.17–0.25)	0.21 (0.17–0.26)	0.21 (0.19–0.23)
		End	0.24 (0.23–0.30)	0.26 (0.21–0.28)	0.20 (0.17–0.22)	0.22 (0.20–0.27)	0.25 (0.23–0.27)	0.24 (0.21–0.25)
	Hard	Begin	0.23 (0.18–0.25)	0.23 (0.21–0.28)	0.19 (0.17–0.21)	0.23 (0.20–0.26)	0.22 (0.15–0.29)	0.23 (0.21–0.26)
		Middle	0.18 (0.14–0.20)	0.20 (0.19–0.25)	0.17 (0.15–0.18)	0.21 (0.17–0.23)	0.18 (0.16–0.26)	0.20 (0.18–0.23)
		End	0.21 (0.20–0.23)	0.22 (0.20–0.26)	0.23 (0.22–0.25)	0.20 (0.18–0.22)	0.25 (0.19–0.29)	0.23 (0.21–0.25)
EMG activity (closing phase)	Soft	Begin	1.35 (1.14–1.57)	1.40 (1.29–1.68)	1.35 (1.0–1.51)	1.52 (1.21–1.70)	1.80 (1.56–2.18)	1.42 (1.26–1.59)
		Middle	1.31 (0.93–1.46)	1.0 (0.92–1.05)	0.84 (0.75–0.97)	1.10 (0.96–1.24)	1.142 (1.0–1.38)	0.92 (0.81–1.02)
		End	0.70 (0.41–1.54)	0.58 (0.34–0.69)	0.46 (0.37–0.57)	0.73 (0.54–0.77)	0.57 (0.49–0.65)	0.34 (0.29–0.41)
	Hard	Begin	1.55 (1.31–1.83)	1.68 (1.27–2.26)	1.77 (1.53–2.28)	1.29 (1.14–1.53)	1.44 (1.08–1.52)	1.92 (1.77–2.02)
		Middle	1.33 (1.02–1.55)	1.166 (1.0–1.48)	1.18 (1.01–1.39)	0.97 (0.83–1.08)	0.85 (0.67–1.0)	1.07 (1.02–1.14)
		End	0.81 (0.52–1.46)	0.66 (0.53–0.74)	0.53 (0.47–0.69)	0.54 (0.35–0.75)	0.38 (0.26–0.46)	0.31 (0.27–0.51)
EMG activity (occlusal phase)	Soft	Begin	0.51 (0.39–1.17)	1.35 (1.24–1.54)	1.41 (0.95–1.46)	1.35 (1.09–1.65)	1.73 (1.49–2.10)	1.37 (1.22–1.56)
		Middle	0.68 (0.39–0.84)	0.94 (0.86–0.99)	0.81 (0.67–0.92)	0.97 (0.81–1.15)	1.09 (0.96–1.32)	0.89 (0.74–1.0)
		End	0.48 (0.34–0.71)	0.55 (0.30–0.64)	0.43 (0.30–0.54)	0.62 (0.50–0.69)	0.52 (0.46–0.61)	0.31 (0.26–0.36)
	Hard	Begin	0.98 (0.50–1.77)	1.63 (1.22–2.09)	1.73 (1.48–2.19)	1.21 (1.04–1.45)	1.31 (0.99–1.44)	1.87 (1.71–1.93)
		Middle	0.77 (0.60–1.17)	1.11 (0.96–1.36)	1.16 (0.98–1.30)	0.87 (0.76–1.0)	0.72 (0.53–0.95)	1.03 (0.98–1.11)
		End	0.61 (0.42–0.86)	0.60 (0.47–0.70)	0.50 (0.44–0.63)	0.42 (0.31–0.55)	0.35 (0.19–0.42)	0.28 (0.24–0.47)

**Table 3:** Summary of the results obtained for each of the outcome parameter during the Kruskal–Wallis H analysis of between-group differences and the Friedman ANOVA and Kendall Coefficient of Concordance analysis of the differences between the three segments of the chewing sequence (beginning, middle, and end) of each of the outcome parameters within each age-group

Outcome parameter	Food	Chewing segment	Between-Group difference^1^	Within-group difference^2^					
				Primary	Early-mixed	Late-mixed	Early-permanent	Late-permanent	Adults
Chewing cycle (n)	Soft	ــ	NS	NA *	NA	NA	NA	NA	Na*
	Hard	ــ	NS	NA *	NA	NA	NA	NA	Na*
Chewing duration (s)	Soft	ــ	NS	NA	NA	NA	NA	NA	Na*
	Hard	ــ	NS	NA	NA	NA	NA	NA	Na*
Chewing rate (Hz)	Soft	ــ	NS	NA *	NA	NA	NA	Na*	Na*
	Hard	ــ	*p* = 0.0419	NA *	NA	NA	NA	Na*	Na*
Vertical amplitude (mm)	Soft	Begin	NS	*p* = 0.0247	*p* = 0.0001	*p* = 0.0273	*p* = 0.0003	*p* = 0.0022	*p* = 0.0007*
		Middle	NS						
		End	NS						
	Hard	Begin	NS	*p* = 0.0017	p = 0.0003	*p* = 0.0202	*p* = 0.0034	*p* = 0.00123	*p* = 0.0007*
		Middle	NS						
		End	NS						
Lateral amplitude (mm)	Soft	Begin	*p* = 0.0181	NS	NS	NS	NS	*p* = 0.0018	p = 0.0273
		Middle	*p* = 0.0063						
		End	*p* = 0.0267						
	Hard	Begin	*p* = 0.0123	NS	NS	*p* = 0.0075	NS	*p* = 0.0075	*p* = 0.0451
		Middle	*p* = 0.0046						
		End	*p* = 0.0004						
Jaw opening velocity (mm/s)	Soft	Begin	NS	NS*	*p* = 0.0136	p = 0.0451	*p* = 0.0082	*p* = 0.0003*	*p* = 0.0136*
		Middle	NS						
		End	NS						
	Hard	Begin	NS	*p* = 0.0136*	*p* = 0.0136	*p* = 0.0451	*p* = 0.0136	*p* = 0.0075*	*p* = 0.0055*
		Middle	NS						
		End	NS						
Jaw-closing velocity (mm/s)	Soft	Begin	NS	*p* = 0.0451	*p* = 0.0012*	*p* = 0.0075	*p* = 0.0055	*p* = 0.0202*	*p* = 0.0451*
		Middle	NS						
		End	NS						
	Hard	Begin	NS	*p* = 0.0006	p = 0.0075*	*p* = 0.0082	*p* = 0.0034	*p* = 0.0004*	*p* = 0.0082*
		Middle	NS						
		End	NS						
Occlusal duration (s)	Soft	Begin	NS	NS*	NS*	NS	NS	NS	NS
		Middle	NS						
		End	NS						
	Hard	Begin	NS	*p* = 0.0075*	*p* = 0.0451*	*p* = 0.0075	NS	NS	NS
		Middle	NS						
		End	NS						
Opening duration (s)	Soft	Begin	NS	NS*	NS	NS	*p* = 0.0451	NS	NS
		Middle	NS						
		End	NS						
	Hard	Begin	*p* = 0.0149	NS*	NS	NS	NS	*p* = 0.0202	NS
		Middle	NS						
		End	*p* = 0.0099						
Closing duration (s)	Soft	Begin	NS	NS	NS	*p* = 0.0451	NS	NS	*p* = 0.0247
		Middle	NS						
		End	NS						
	Hard	Begin	NS	NS	NS	*p* = 0.0012	NS	NS	NS
		Middle	NS						
		End	NS						
EMG activity (closing phase)	Soft	Begin	*p* = 0.0474	NS	*p* = 0.0001	*p* = 0.0001	*p* = 0.0001	*p* = 0.0001*	*p* = 0.0001*
		Middle	*p* = 0.0232						
		End	*p* = 0.0110						
	Hard	Begin	*p* = 0.0168	NS	*p* = 0.0001	*p* = 0.0001	*p* = 0.0001	*p* = 0.0002*	*p* = 0.0001*
		Middle	p = 0.0075						
		End	*p* = 0.0054						
EMG activity (occlusal phase)	Soft	Begin	p = 0.0202	NS	*p* = 0.0001	*p* = 0.0001	*p* = 0.0007	*p* = 0.0001*	*p* = 0.0001*
		Middle	*p* = 0.0309						
		End	*p* = 0.0241						
	Hard	Begin	*p* = 0.0049	NS	*p* = 0.0001	*p* = 0.0001	*p* = 0.0001	*p* = 0.0012*	*p* = 0.0001*
		Middle	*p* = 0.0039						
		End	*p* = 0.0108						

(1) Between-group differences were analyzed with Kruskal–Wallis H test with pairwise post hoc comparison using Dunn-Bonferroni correction. (2) Friedman ANOVA and Kendall Coefficient of Concordance test were used to analyze within age-group differences of the three segments of the chewing sequence (beginning, middle, and end) of each of the outcome parameter. Wilcoxon Matched Pairs Test was used to analyze the within-group differences between the soft and hard viscoelastic food at the beginning of the chewing sequence; significant results indicated by asterisk (*) placed on both the two types of food. The significance level was set at 0.05 for all the statistical tests. NS indicates a *p* value > 0.05 and (NA) indicates no applicable within-group difference in the outcome variable with the chewing progression

### Chewing sequence

**Fig. 2:**
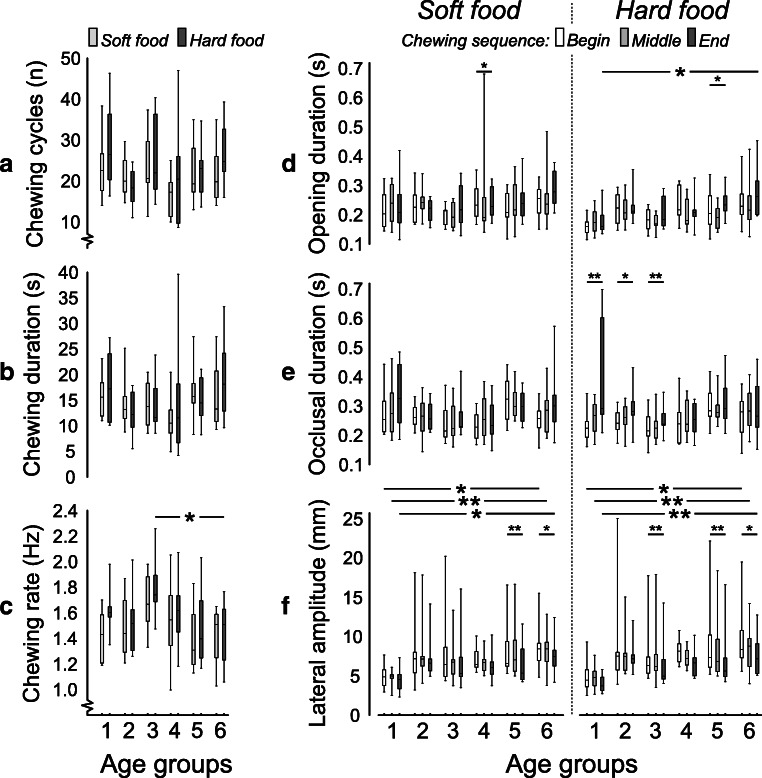
Box plots of the number of chewing cycles (**a**), chewing sequence duration (**b**), chewing rate (**c**), jaw-opening duration (**d**), occlusal duration (**e**), and lateral jaw movement amplitude (**f**) for the soft and hard viscoelastic test food models performed by each of the six age-groups: primary (1), early-mixed (2), late-mixed (3), early-permanent (4), and late-permanent (5) dentition groups and adults (6). Significant results of between-group differences were denoted (* = *p* ˂ 0.05; ** = *p* ˂ 0.01; *** = *p* ˂ 0.001)

There were no significant differences in the number of chewing cycles and duration of the chewing sequence between the children and the adult group neither while eating hard nor while eating soft food (Fig. 2a and b). However, the children in the late-mixed dentition group exhibited a higher chewing rate compared with adults during eating hard food (*p* = 0.044) but not soft food (Fig. 2c).

It was observed that all the children groups chewed both the hard and soft food with an equal number of chewing cycles and chewing duration. However, the children in the primary dentition group and the adult group chewed with a higher number of chewing cycles, while eating hard food as compared with soft food (*p* ˂ 0.01). Moreover, the adult group chewed hard food for a longer duration than soft food (*p* = 0.009).

### Jaw kinematics

The velocity of the jaw opening and jaw-closing did not differ between the children and the adult groups. There were also no differences in the vertical jaw amplitude between the children and the adult groups, neither while eating hard nor while eating soft food. However, the lateral jaw amplitude was significantly shorter in the primary dentition group compared with adults while eating both hard and soft food throughout the chewing sequence (*p* ˂ 0.05; Fig. 2f). The jaw-opening duration was significantly shorter in the primary dentition group as compared with the adult group, while eating hard food, at the beginning and the end of the chewing sequence (*p* = 0.015 and *p* = 0.009 for the two chewing segments, respectively; Fig. 2d). However, no between-group differences were found for the jaw-closing and occlusal duration while eating neither hard nor soft food.

The late-permanent dentition group and the adult group showed slower jaw-opening velocity and jaw-closing velocity while eating soft food than while eating hard food (*p* ˂ 0.03). Also, the jaw-opening velocity in the primary dentition group and the jaw-closing velocity in the early-mixed dentition group were slower while eating soft food (*p* = 0.022 and *p* = 0.007, respectively). Both the vertical and lateral jaw amplitudes remained the same for both the food types. Further, the jaw-opening duration in the primary dentition group was shorter while eating hard food (*p* = 0.037); yet there were no differences in the jaw-closing duration in any of the groups. The occlusal duration was also significantly shorter in the primary and the early-mixed dentition while eating hard food than soft food (*p* = 0.001 and *p* = 0.022 for the two age-groups, respectively; Fig. 2e).

The primary dentition group showed no differences in the jaw-opening velocity with the progression of chewing sequence (beginning, middle, and end) while eating soft food. However, the jaw-opening velocity decreased with the progression of the chewing sequence, while eating both the hard and the soft food for all the other groups (*p* ˂ 0.05). Similarly, the jaw-closing velocity also decreased with the progression of the chewing sequence, while eating both hard and soft food for all the groups (*p* ˂ 0.05). There was a decrease in the vertical jaw amplitude with the progression of the chewing sequence while eating both the hard and the soft food for all the groups. Whereas, the lateral jaw amplitude in the late permanent dentition and the adult group decreased with the progression of chewing sequence while eating both hard and soft food (*p* ˂ 0.05). However, the late-mixed dentition group showed a decrease in lateral jaw amplitude with the progression of chewing sequence while eating hard food only (*p* = 0.008). The occlusal duration in the primary-, early-, and late-mixed dentition groups increased with the progression of chewing sequence while eating hard food only (*p* = 0.008, *p* = 0.045, and *p* = 0.008 for the age-groups, respectively).

### EMG activity

**Fig. 3:**
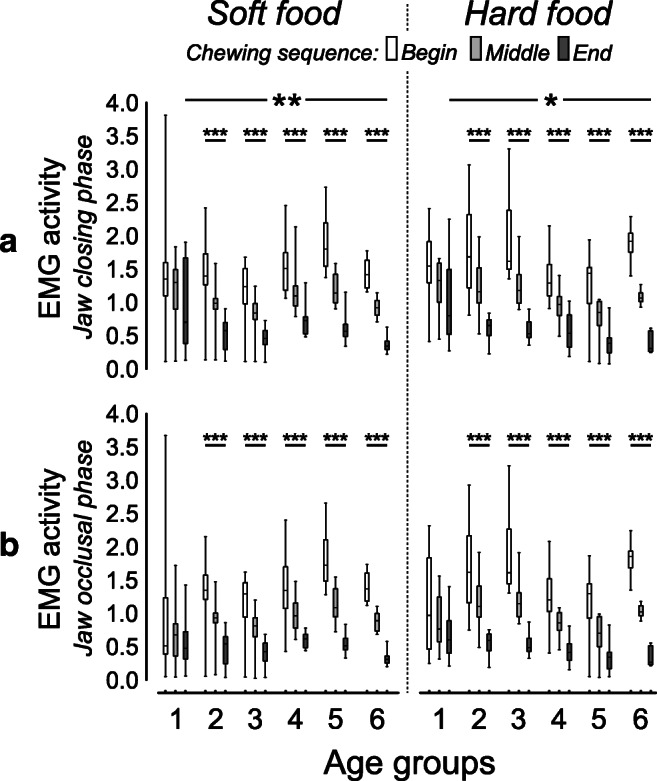
Box plots of the normalized masseter muscle activity during jaw-closing (**A**), and jaw-occlusal (**B**) phases for the soft and hard viscoelastic test food models performed by each of the six age-groups: primary (1), early-mixed (2), late-mixed (3), early-permanent (4), and late-permanent (5) dentition groups and adults (6). Significant results of between-group differences and for the chewing progression within-group difference were denoted (* = *p* ˂ 0.05; ** = *p* ˂ 0.01; *** = *p* ˂ 0.001)

The EMG activity of the masseter muscle during the jaw-closing phase was significantly higher in the primary dentition group compared with the adult group, while eating both hard and soft food (*p* = 0.002 and *p* = 0.025 for the two types of food, respectively; Fig. 3). The early-permanent dentition group showed higher EMG activity during the occlusal phase compared with the adult group while eating soft food only (*p* = 0.009). However, these differences in the EMG activity of the masseter muscles both during the jaw-closing and the occlusal phases were evident only at the end of the chewing sequence.

The EMG activity in the adult group was higher while eating hard food than soft food during both the jaw-closing and the occlusal phases (*p* = 0.005 for the two phases). Further, the EMG activity in the late-permanent dentation group was lower while eating the hard food than the soft food (*p* = 0.047 and *p* = 0.037 for the closing and occlusal phases, respectively). However, all other age-groups showed no differences in the EMG activity while eating both the hard and the soft food.

The EMG activity during the jaw-closing and occlusal phases decreased with the progression of the chewing sequence while eating both hard and soft food for all the groups (*p* ˂ 0.05). However, the primary dentition group showed no differences in the EMG activity with the progression of the chewing sequence, while eating both the hard and soft food.

## Discussion

During childhood, the sensorimotor control governing the jaw motor actions must adapt to substantial morphological changes due to growth. Studies on chewing behavior suggest that children have a characteristic pattern that differs from adults and that certain movement parameters change with age. However, current knowledge about the age-related changes in the sensorimotor system controlling the jaw motor actions is yet to be fully elucidated [17]. Therefore, the current study investigated the age-related changes in jaw kinematics and jaw muscle activity while eating viscoelastic food with different hardness. The results revealed that children with primary dentition had shorter lateral jaw movement and higher muscle activity at the end of the chewing sequence, in comparison with adults. While the chewing sequence duration was not affected by food hardness in the primary dentition group, yet they increased the number of chewing cycles and the chewing rate while eating hard food compared with soft. However, this group of children failed to adapt (increase) their jaw muscle activity to food hardness similar to children in the early-mixed, late-mixed, and early-permanent dentition groups (3–14 years). It was also observed that children in the late-permanent dentition stage (15 to 17 years) were capable of performing adult-like chewing behavior. To our best knowledge, this is the first study to evaluate masticatory jaw kinematics and muscle activity in growing healthy children in comparison with adults.

Variation in food properties such as texture, size, and weight can influence chewing behavior [21]. Most studies used natural food to investigate the adaptation of chewing behavior to food hardness. However, it is difficult to distinguish if the response of the masticatory system to the natural food is due to food hardness or due to other textural properties (such as elasticity, plasticity, stickiness, brittleness to name a few) [21]. Therefore, in order to avoid any variations in chewing parameters caused by food inconsistencies, an edible viscoelastic food with controlled properties was developed [10, 22]. In the current study, the same viscoelastic food was produced into two grades of hardness, which had the same rheological properties [10, 22].

### Age-related differences in chewing behavior

While some studies on healthy children have shown that the number of chewing cycles and the duration of chewing sequence decreased with age, others showed an increase with age (for review see [17]). However, in the current study, there was no difference in the number of chewing cycles and the duration of the chewing sequence between children groups and adults. Further, our results showed age-group differences in the lateral jaw amplitude and jaw opening duration. Both the lateral jaw amplitude and jaw-opening duration were shorter in the primary dentition group compared with the adults. Previous studies showed that the jaw opening duration during chewing is shorter in healthy children than adults [23–26], though other studies reported conflicting results concerning the influence of age on the lateral jaw movement during chewing behavior [23, 26, 27]. In particular, two studies showed that the lateral jaw amplitude is similar between children and adults [26, 27], whereas another study found that children eat with shorter lateral jaw amplitude compared with adults [23]. However, we believe that the shorter lateral jaw movement in the primary dentition group is due to differences in the jaw dimensions in the children compared with adults. The shorter lateral jaw movement might also explain the shorter jaw-opening duration observed in the primary dentition group than adults.

During the progression of the chewing sequence, the particle size is reduced, and the food is pulverized, which typically leads to a reduction in jaw muscle activity [9, 10, 28]. However, in the current study, it was observed that children in the primary dentition group (3–6 years) did not decrease their muscle activity with the progression of chewing sequence as compared with adults. This observation in the children with primary dentition could be attributed to the histological features of the jaw-closing muscles and the food physical properties [29]. It was previously shown that the fiber diameter of the masseter muscle in adults is almost twice the size of the masseter muscle in young children (3–7 years) [29]. The differences in fiber diameter between young children and the adult may indicate differences between children and adults in regard to the contractile properties of the masseter muscle, such as power and strength [30]. It has been suggested that the unchanged jaw muscle activity with the progression of the chewing sequence can be due to the physical properties of the food [31]. It was shown that jaw muscle activity is influenced by food adhesiveness and cohesiveness [31]. It was also suggested that food morsels with higher cohesion and adhesion properties require more chewing efforts to break the food down into smaller pieces [32] and that the mouthful volume of the food morsel can influence appropriate food bolus formation [33]. In the current study, as the food boli formation was not evaluated, yet it was observed that all the participants were able to chew and swallow the test food models with no visible difficulties. Based on these observations from the previous studies and the current study, a number of speculations can be drawn. For example, although children with the primary dentition were able to eat the viscoelastic food with a similar number of cycles and duration as adults; yet we assume that the volume of the food bolus formed before swallowing was “quite a mouthful.” The bolus thus formed would have needed to be divided into smaller boli before swallowing, leading to no change in the EMG activity with the progression of chewing sequence. Also, it could be assumed that children end up swallowing larger food particles compared with adults. Taken all together, the age-related histological differences in the masseter muscle, the cohesion and adhesion properties of the test food models, and the mouthful volume of test food models may explain the unchanged jaw muscle activity with the chewing progression in the primary dentition group compared with adults.

### Age-related changes in food hardness adaptation during natural chewing

Adults, in the current study, reliably increased both the number of chewing cycles and chewing sequence duration in relation to food hardness. Specifically, the adults showed a higher number of chewing cycles and longer chewing sequence duration while eating hard food than soft food. However, the children groups (3–17 years) did not show any such differences in the number of chewing cycles and/or chewing sequence duration between different food hardness. It has been suggested that a lesser number of chewing cycles or shorter chewing sequence duration may not be good indicators of chewing performance [34]. A good masticatory performance could be assessed as the “right” texture of the boli and the right time where the food has been adequately pulverized, and an “ideal” agglomeration of the bolus is achieved. Food properties such as size, hardness, elasticity, and dryness of the food morsel could affect the pulverization or the agglomeration of the food morsel [10, 35]. However, in the present study, it was observed that while the adults were able to adapt to the food hardness by increasing the number of chewing cycles and the duration of chewing sequence while eating hard food, none of the children groups showed any such behavior.

The children groups (3–14 years) did not adapt their jaw muscle activity to food hardness at the beginning of the chewing sequence. Typically, during the initial tooth/food contact, a major fraction of the jaw muscle activity (also known as additional muscle activity) is used to overcome the resistance of the food [36]. Therefore, in people with natural dentition, a typical observation is an increase in the EMG activity during the initial tooth/food contact, and this increase is more prominent while chewing hard food than soft food [9–13]. Hence, there is an adaptation of jaw muscle activity according to the specifics of the task demands in particularly chewing different types of food. The current study suggests that this adaptation to food hardness is “immature” in the abovementioned children groups.

The occlusal duration of the chewing cycle while eating hard food increased with the progression of the chewing sequence in children groups 3–11 years. A series of studies have established that periodontal mechanoreceptors (PMRs) signal vital information about the initial tooth/food contact [8, 9]. This vital information is used by the central nervous system to execute jaw motor actions. It was observed that when the sensory information from the PMRs is perturbed (such as due to local anesthesia or lack of PMRs as in implants), the participants showed impaired chewing behavior compared with people with natural dentition [9, 12, 13, 37–44]. Specifically, these patients demonstrated an increase in the occlusal cycle duration while eating hard food [9, 12, 13, 37–41]. It was also shown that patients with dental implant showed signs of weaker muscle adaptation with the increased food hardness [9]. Further, the dental implant patients showed poor adaptation of the jaw muscle activity with the progression of the chewing sequence (i.e., when the food is pulverized) [9]. It was also suggested that the lack of adaptation of the jaw muscle activity to the food hardness observed in these groups of individuals was mostly prominent at the beginning of the chewing sequence. While children do not have similar sensory disruption from the PMRs, yet they showed an increased occlusal duration and failure to adapt the jaw muscle activity to food hardness. This observation could be supported by the histological findings of the PMRs from the animal studies. Accordingly, the histological development of the PMRs during the changing dentition from childhood to adulthood is unknown in humans. Animal studies, on the other hand, showed that the primary teeth in cats had reduced PMRs density in comparison with permanent teeth [45]. Further, an association was shown between the PMRs morphological changes and tooth eruption and also the development of teeth occlusion [46]. If the results of animal studies can be extrapolated into humans, it may be speculated that there is inadequate (immature) sensory input from the PMRs in the primary/mixed dentition compared with the permanent dentition. This inadequate sensory input resulted in increased occlusal duration as observed in the current study, where the children groups (3–11 years) took a longer time to collect the sensory information from the PMRs. The longer occlusal time was also shown in healthy young adults during a sudden deprivation of sensory input from periodontium due to anesthesia [13]. Recently, it was observed that children between 3 and 14 years showed higher and more variable forces when manipulating a food morsel between their front teeth [47]. This increased force magnitude and variability were attributed to immature sensorimotor control during an oral fine motor task.

### Study limitations and strengths

Methodological considerations are quite evident and should be acknowledged in scientific research. The underpowered sample of the current study is one such methodological constraint. Thus, to minimize the likelihood of increasing false-positive results with statistical analysis due to the underpowered sample, nonparametric tests were used, which are more conservative than the more robust parametric tests. Another methodological limitation was the number of dropouts from the present study. In this study, we followed an elaborate procedure to recruit the children. Accordingly, all the children who fulfilled the inclusion/exclusion criteria were approached by the attending clinician. The clinician gave the selected children and their legal representatives oral information about the study. Once they agreed to participate, they were approached by the principal investigator (NA). The laboratory, where the experiment took place, was customized to be more “child-friendly.” The children especially were allowed enough time to familiarize and be comfortable with the lab environment before the start of the study. However, a relatively large number of younger children (22 children; mean age 5.8 years, SD: 2.5) showed signs of apprehension; therefore, we were unsure if they could perform the task legibly. Thus, only the remaining children who cooperated and performed the experiment in a reliable manner were finally recruited for the data collection. Future studies may include a separate familiarization session to expose and acquaint the children to the lab environment and the experimental protocol. This was not feasible to be applied in the current study due to the restricted timeframe of children’s parents/legal guardians. Further, previous literature indicated that the development of chewing parameters such as bite force, jaw muscle activity, and jaw kinematics was mainly influenced by the dentition status (for review see [17]). Thus, in the current study, the participating children were categorized according to their dental status, which illustrates five transitory stages of dental eruption. Nonetheless, we believe that the number of occluding contacts is another variable in chewing development. However, due to the relatively small sample size in the current study, this was not explored, and future research may be directed to investigate the influence of the number of occluding teeth or sex, particularly, on the development of chewing behavior in healthy children.

### Clinical implication

While growth reference values for body weight, length, and head circumference are available, such reference values for the development of the chewing behavior are lacking. Hence, the current study aimed to establish the developmental milestones of chewing behavior in healthy children. The obtained knowledge will help us to identify children who are at risk of chewing impairment due to several orofacial dysfunctions. A prime example of such dysfunctions is dental/skeletal malocclusion in children with orthodontic treatment needs. It was suggested that children with malocclusion were shown to have reduced bite forces and poor masticatory performance than children with normal occlusion [48]. Therefore, identifying the developmental milestones of chewing behavior in healthy children will help us compare, diagnose, and evaluate the success of the orthodontic/orthognathic treatment in children with malocclusions.

## Conclusions

Overall, it seems that jaw motor functions in children as young as 3–6 years is quite competent in performing basic chewing function similar to adults. Yet, there were differences in the anticipation/adaption of jaw muscle activity and jaw kinematics to food hardness. These subtle, yet significant, differences could be attributed to the anatomy of the masticatory system, such as jaw dimensions. Further, the differences can also be attributed to the inadequate (immature) sensory input from the PMRs, while the teeth shift from primary to mixed dentition and finally to permanent dentition with a complete tooth root formation and occlusal table. Recently, we have suggested that the contribution of PMRs to jaw muscle activity is about 20% during chewing [13]. We think that because of the reduced PMRs density in children compared with adults, the contribution of PMRs in jaw muscle activity is rather affected in young children, which leads to the differences in chewing behavior.

## References

[1] Naka O, Anastassiadou V, Pissiotis A (2014). Association between functional tooth units and chewing ability in older adults: a systematic review. Gerodontology.

[2] Chen J (2009). Food oral processing—a review. Food Hydrocoll.

[3] Watson S, McGowan L, McCrum LA, Cardwell CR, McGuinness B, Moore C, Woodside JV, McKenna G (2019). The impact of dental status on perceived ability to eat certain foods and nutrient intakes in older adults: cross-sectional analysis of the UK National Diet and Nutrition Survey 2008-2014. Int J Behav Nutr Phys Act.

[4] Sheiham A, Steele J (2001). Does the condition of the mouth and teeth affect the ability to eat certain foods, nutrient and dietary intake and nutritional status amongst older people?. Public Health Nutr.

[5] Brennan DS, Spencer AJ, Roberts-Thomson KF (2008). Tooth loss, chewing ability and quality of life. Qual Life Res.

[6] Dellow PG, Lund JP (1971). Evidence for central timing of rhythmical mastication. J Physiol.

[7] Sessle BJ (2011). Face sensorimotor cortex. Breathe Walk and Chew; The Neural Challenge: Part II.

[8] Lund JP (1991). Mastication and its control by the brain stem. Crit Rev Oral Biol Med.

[9] Grigoriadis A, Johansson RS, Trulsson M (2011). Adaptability of mastication in people with implant-supported bridges. J Clin Periodontol.

[10] Peyron MA, Lassauzay C, Woda A (2002). Effects of increased hardness on jaw movement and muscle activity during chewing of visco-elastic model foods. Exp Brain Res.

[11] Grigoriadis A, Johansson RS, Trulsson M (2014). Temporal profile and amplitude of human masseter muscle activity is adapted to food properties during individual chewing cycles. J Oral Rehabil.

[12] Grigoriadis A, Trulsson M (2018). Excitatory drive of masseter muscle during mastication with dental implants. Sci Rep.

[13] Grigoriadis A, Kumar A, Åberg MK, Trulsson M (2019) Effect of sudden deprivation of sensory inputs from periodontium on mastication. Front Neurosci 13. 10.3389/fnins.2019.01316 10.3389/fnins.2019.01316 PMC6914695 31920486

[14] Peyron MA, Woda A, Bourdiol P, Hennequin M (2017). Age-related changes in mastication. J Oral Rehabil.

[15] Peyron M-A, Blanc O, Lund JP, Woda A (2004). Influence of age on adaptability of human mastication. J Neurophysiol.

[16] Avivi-Arber L, Sessle BJ (2018). Jaw sensorimotor control in healthy adults and effects of ageing. J Oral Rehabil.

[17] Almotairy N, Kumar A, Trulsson M, Grigoriadis A (2018). Development of the jaw sensorimotor control and chewing - a systematic review. Physiol Behav.

[18] Wickwire NA, Gibbs CH, Jacobson AP, Lundeen HC (1981). Chewing patterns in normal children. Angle Orthod.

[19] Kumar A, Castrillon E, Svensson KG, Baad-Hansen L, Trulsson M, Svensson P (2015). Effects of experimental craniofacial pain on fine jaw motor control: a placebo-controlled double-blinded study. Exp Brain Res.

[20] Kumar A, Castrillon E, Svensson P (2015). Can Experimentally Evoked Pain in the Jaw Muscles or Temporomandibular Joint Affect Anterior Bite Force in Humans?. J Oral Facial Pain Headache.

[21] Woda A, Foster K, Mishellany A, Peyron MA (2006). Adaptation of healthy mastication to factors pertaining to the individual or to the food. Physiol Behav.

[22] Lassauzay C, Peyron MA, Albuisson E, Dransfield E, Woda A (2000). Variability of the masticatory process during chewing of elastic model foods. Eur J Oral Sci.

[23] Kubota N, Hayasaki H, Saitoh I, Iwase Y, Maruyama T, Inada E, Hasegawa H, Yamada C, Takemoto Y, Matsumoto Y, Yamasaki Y (2010). Jaw motion during gum-chewing in children with primary dentition. Cranio.

[24] Snipes WB, Throckmorton GS, Buschang PH (1998). Normal masticatory function of girls and young women: mandibular masticatory movements. Am J Hum Biol.

[25] Yamada-Ito C, Saitoh I, Yashiro K, Inada E, Maruyama T, Takada K, Iwasaki T, Hayasaki H, Yamasaki Y (2013). Smoothness of molar movement during gum chewing in children with primary dentition. Cranio.

[26] Kiliaridis S, Karlsson S, Kjellberg H (1991). Characteristics of masticatory mandibular movements and velocity in growing individuals and young adults. J Dent Res.

[27] Papargyriou G, Kjellberg H, Kiliaridis S (2000). Changes in masticatory mandibular movements in growing individuals: a six-year follow-up. Acta Odontol Scand.

[28] Veyrune JL, Lassauzay C, Nicolas E, Peyron MA, Woda A (2007). Mastication of model products in complete denture wearers. Arch Oral Biol.

[29] Österlund C, Thornell L-E, Eriksson P-O (2011). Differences in fibre type composition between human masseter and biceps muscles in young and adults reveal unique masseter fibre type growth pattern. Anat Rec Adv Integr Anat Evol Biol.

[30] Bakke M, Holm B, Jensen BL, Michler L, Möller E (1990). Unilateral, isometric bite force in 8-68-year-old women and men related to occlusal factors. Scand J Dent Res.

[31] Iguchi H, Magara J, Nakamura Y, Tsujimura T, Ito K, Inoue M (2015). Changes in jaw muscle activity and the physical properties of foods with different textures during chewing behaviors. Physiol Behav.

[32] Kohyama K, Sawada H, Nonaka M, Kobori C, Hayakawa F, Sasaki T (2007). Textural evaluation of Rice cake by chewing and swallowing measurements on human subjects. Biosci Biotechnol Biochem.

[33] Goto T, Nakamich A, Watanabe M, Nagao K, Matsuyama M, Ichikawa T (2015). Influence of food volume per mouthful on chewing and bolus properties. Physiol Behav.

[34] Slavicek G (2010). Human mastication. Int J Stomatol Occlusion Med.

[35] van der Bilt A, Engelen L, Pereira LJ, van der Glas HW, Abbink JH (2006). Oral physiology and mastication. Physiol Behav.

[36] Ottenhoff FA, van der Bilt A, van der Glas HW, Bosman F (1992). Control of elevator muscle activity during simulated chewing with varying food resistance in humans. J Neurophysiol.

[37] Svensson KG, Trulsson M (2009). Regulation of bite force increase during splitting of food. Eur J Oral Sci.

[38] Johansson AS, Svensson KG, Trulsson M (2006). Impaired masticatory behavior in subjects with reduced periodontal tissue support. J Periodontol.

[39] Kumar A, Castrillon E, Trulsson M, Svensson KG, Svensson P (2017). Fine motor control of the jaw following alteration of orofacial afferent inputs. Clin Oral Investig.

[40] Svensson KG, Grigoriadis J, Trulsson M (2013). Alterations in intraoral manipulation and splitting of food by subjects with tooth- or implant-supported fixed prostheses. Clin Oral Implants Res.

[41] Grigoriadis J, Trulsson M, Svensson KG (2016). Motor behavior during the first chewing cycle in subjects with fixed tooth- or implant-supported prostheses. Clin Oral Implants Res.

[42] Kumar A, Tanaka Y, Takahashi K, Grigoriadis A, Wiesinger B, Svensson P, Trulsson M (2019). Vibratory stimulus to the masseter muscle impairs the oral fine motor control during biting tasks. J Prosthodont Res.

[43] Kumar A, Grigoriadis J, Trulsson M, Svensson P, Svensson KG (2015). Effects of short-term training on behavioral learning and skill acquisition during intraoral fine motor task. Neuroscience..

[44] Grigoriadis J, Kumar A, Svensson P, Svensson KG, Trulsson M (2017). Perturbed oral motor control due to anesthesia during intraoral manipulation of food. Sci Rep.

[45] Miki K, Honma S, Ebara S, Kumamoto K, Murakami S, Wakisaka S (2015). Changes in the distribution of periodontal nerve fibers during dentition transition in the cat. PLoS One.

[46] Maeda T, Ochi K, Nakakura-Ohshima K, Youn SH, Wakisaka S (1999). The Ruffini ending as the primary mechanoreceptor in the periodontal ligament: its morphology, cytochemical features, regeneration, and development. Crit Rev Oral Biol Med.

[47] Almotairy N, Kumar A, Noirrit-Esclassan E, Grigoriadis A (2020). Developmental and age-related changes in sensorimotor regulation of biting maneuvers in humans. Physiol Behav.

[48] Magalhaes IB, Pereira LJ, Marques LS, Gameiro GH (2010). The influence of malocclusion on masticatory performance. A systematic review. Angle Orthod.

